# The association between glycometabolism and nonalcoholic fatty liver disease in patients with obstructive sleep apnea

**DOI:** 10.1007/s11325-018-1744-1

**Published:** 2018-10-22

**Authors:** Haibo Ding, Jie-feng Huang, Han-Sheng Xie, Bi-Ying Wang, Ting Lin, Jian-Ming Zhao, Qi-Chang Lin

**Affiliations:** 1 Fujian Provincial Sleep-disordered Breathing Clinic Center, Fuzhou, People’s Republic of China; 2 Laboratory of Respiratory Disease of the Fujian Medical University, Fuzhou, People’s Republic of China; 3 Department of Respiratory and Critical Care Medicine, the First Affiliated Hospital of Fujian Medical University, No. 20, Chazhong road, Taijiang district, Fuzhou, Fujian Province 350005 People’s Republic of China

**Keywords:** Obstructive sleep apnea, Glycometabolism, NAFLD, Insulin resistance

## Abstract

**Purpose:**

Growing evidence has revealed that nonalcoholic fatty liver disease (NAFLD) is associated with type 2 diabetes. This study aimed to assess the association between glycometabolism and NAFLD in patients with obstructive sleep apnea (OSA).

**Methods:**

Patients with suspected OSA were enrolled consecutively and then underwent polysomnography, liver ultrasound, and biochemical measurements. Logistic regressions were used to identify factors associated with NAFLD.

**Results:**

In total, 415 patients were included. The prevalence of NAFLD in the non-OSA, mild OSA, moderate OSA, and severe OSA groups was 37.21%, 69.09%, 68.34%, and 78.08%, respectively. Stepwise logistic regression suggested that percentage of total sleep time spent with oxygen saturation of < 90% (TS90), lowest oxygen saturation (LaSO_2_), and homeostatic model assessment of insulin resistance (HOMA-IR) were independently associated with NAFLD in all subjects, after adjusting for confounders (odds ratio [OR] = 1.037, *p* = 0.014; OR = 1.056, *p* = 0.004; OR = 0.732, *p* = 0.009; respectively). TS90, LaSO_2_, and HOMA-IR were also independent predictors for NAFLD in patients with mild and moderate OSA, whereas TS90, LaSO_2_, and ODI were independent predictors for NAFLD in patients with severe OSA.

**Conclusions:**

There is a relationship between OSA and NAFLD, and the combination of disordered glycometabolism and intermittent hypoxia may act as a “two hit” mechanism to promote the development of NAFLD. Furthermore, intermittent hypoxia alone was an independent predictor for NAFLD in severe OSA patients.

## Introduction

Nonalcoholic fatty liver disease (NAFLD) is the most common chronic liver disease that affects approximately 20 to 30% of the general adult population and is a significant public health problem [1–3]. NAFLD comprises a wide spectrum of liver disorders, including nonalcoholic fatty liver (NAFL), nonalcoholic steatohepatitis (NASH), hepatic fibrosis, cirrhosis, and hepatocellular carcinoma, and is closely linked to obesity, diabetes mellitus (DM), atherosclerosis, cardiovascular disease, and chronic kidney disease, thus representing a substantial clinical and economic burden [4].

Emerging evidence shows that NAFLD is tightly correlated with insulin resistance, diabetes, and other metabolic disorders in non-obese individuals [1]. A “two hit” hypothesis has been put to describe the typical pathogenesis of NAFLD [5]. The “first hit” is excess deposition of adiposity inside hepatic cells, which has been linked to insulin resistance [6]. The “second hit” is increased oxidative stress and enhanced lipid peroxidation, following necrosis and apoptosis of the hepatocyte [7].

Obstructive sleep apnea (OSA) is characterized by repetitive collapse of upper airway during sleep, causing chronic intermittent hypoxia and sleep fragmentation, which lead to uncontrolled lipid peroxidation, hypoxia-reoxygenation injury, and trigger systemic inflammation. There is mounting evidence suggests that OSA is a significant risk factor for glycometabolism disorders [8, 9]. Patients with OSA often exhibit both insulin resistance and oxidative stress, suggesting that these “two hits” contribute to the severity and progression of NAFLD. Emerging evidence suggests that the recurrent episodes of nocturnal hypoxia experienced by patients with OSA are associated with NAFLD, and that continuous positive airway pressure may offer a potential therapeutic option for halting the deterioration of liver disease and reducing the incidence of liver disease [10, 11]. However, the exact mechanisms underlying the effects of OSA on NAFLD remain unclear.

Therefore, based on the concept of the “two hit” hypothesis, the goal of this study was to identify potential interactions between glycometabolism, sleep apnea-induced hypoxia, and NAFLD.

## Methods

### Patients

Consecutive subjects who were referred to our sleep department from January 2016 to January 2018 for sleep disorders such as excessive daytime sleepiness, snoring, and witnessed apnea were recruited. Prior to the start of the study, written consent was obtained from all subjects, and the study was approved by the local institutional ethics committee. None of the patients had been diagnosed with or treated for OSA previously. Information was collected on all of the patients’ sleep symptoms, alcohol consumption, smoking, and current use of medications. Patients with a history of a disease that could lead to pathological fractures, those who had taken any drugs known to influence bone metabolism or the endocrine system, and those with acute inflammatory disease, hyperparathyroidism, chronic kidney disease, or any other conditions that could affect bone metabolism were excluded.

### Anthropometric and biochemical measurements

The body mass index (BMI) was calculated by dividing weight in kilograms by height in meters squared (kg/m^2^). Neck circumference was measured midway between the lower margin of the last palpable rib and the top of the ileal crest, and waist circumference was measured at the level of the umbilicus. Blood pressure was gauged using a standard mercury manometer with a 1-min interval after a 5-min rest. The average value from two readings was used for the analyses.

### Polysomnography

Overnight polysomnography (P Series Sleep System; Compumedics, Melbourne, Australia) was performed to record the following parameters from 22:00 to 06:00: electroencephalography, electrooculography, electromyography, airflow by nasal, and oral thermistors, respiratory effort by thoracic and abdominal impedance belts, and oxyhemoglobin saturation by fingertip pulse oximetry. Sleep staging was scored manually according to the criteria of the American Academia of Sleep Medicine published in 2012 [12]. Obstructive apnea was defined as a decrease in airflow corresponding to 90% or less of baseline airflow for ≥ 10 s. Obstructive hypopnea was defined as a ≥ 30% decrease in airflow lasting at least 10 s with a ≥ 4% decrease in the oxyhemoglobin saturation. The apnea-hypopnea index (AHI) was defined as the total number of apnea and hypopnea events per hour of sleep. The oxygen desaturation index (ODI) was defined as the number of decreases in the oxygen saturation by ≥ 4% per hour of sleep time. Other polysomnographic parameters including the lowest O_2_ saturation (LaSO_2_) and the percentage of total sleep time spent with an oxygen saturation of < 90% (TS90) were also recorded. The patients were divided into four groups depending on the severity of their OSA, based on the AHI thresholds: (I) non-OSA (< 5 events/h), (II) mild OSA (5–14.9 events/h), (III) moderate OSA (AHI 15–29.9 events/h), and (IV) severe OSA (AHI > 30 events/h) (Table 1).

**Table 1:** Comparison of clinical characteristics, laboratory parameters, and polysomnographic parameters according to OSA severity

	Control	Mild	Moderate	Severe	*p*
*n*	43	55	98	219	
Age (years)	46.08 ± 16.83	50.55 ± 11.78	47.90 ± 11.99	49.01 ± 29.91	0.808
Male sex (%)	61.36	67.24	84.00	84.51	0.002
**NA**FLD, number (%)	16 (37.21)	38 (69.09)	67 (68.34)	171 (78.08)	0.000
BMI (kg/m^2^)	24.09 ± 3.67	25.77 ± 3.19	26.38 ± 6.81	27.92 ± 6.25	0.000
Neck circumference (cm)	34.86 ± 8.83	34.77 ± 11.02	37.44 ± 8.70	39.07 ± 8.42	0.002
Waist circumference (cm)	84.70 ± 22.71	85.81 ± 25.41	90.86 ± 20.91	95.74 ± 21.17	0.002
AHI	2.46 ± 1.68	9.72 ± 3.22	21.70 ± 4.08	54.94 ± 16.84	0.000
ODI	4.93 ± 1.24	7.71 ± 5.07	15.37 ± 6.98	46.48 ± 21.02	0.000
LaSO_2_ (%)	95.45 ± 7.65	82.49 ± 8.73	76.97 ± 11.42	66.97 ± 13.95	0.000
mean SpO_2_ (%)	95.89 ± 1.26	95.02 ± 2.11	94.30 ± 2.04	89.97 ± 7.97	0.000
ESS score	5.43 ± 4.52	7.72 ± 4.83	7.51 ± 5.63	10.03 ± 6.03	0.000
AHTI	2.00 ± 1.21	3.36 ± 1.71	8.52 ± 3.14	26.61 ± 12.21	0.000
HbA1c (%)	1.76 ± 2.71	1.94 ± 2.82	2.31 ± 3.11	2.96 ± 3.24	0.032
Fasting glucose (mmol/L)	5.17 ± 1.59	5.07 ± 1.65	5.32 ± 1.31	5.47 ± 1.98	0.389
Insulin (mmol/L)	38.86 ± 44.24	50.73 ± 48.25	55.88 ± 49.91	76.18 ± 45.78	0.007
TC (mmol/L)	4.58 ± 1.33	4.71 ± 1.54	4.75 ± 1.24	5.13 ± 5.38	0.738
TG (mmol/L)	1.56 ± 1.13	1.71 ± 1.08	1.61 ± 1.13	3.98 ± 1.23	0.614
LDL-c (mmol/L)	2.81 ± 1.04	3.13 ± 1.35	3.05 ± 0.99	3.03 ± 1.21	0.578
HDL-c (mmol/L)	1.21 ± 0.50	1.11 ± 0.43	1.14 ± 0.39	1.07 ± 0.70	0.463
HOMA-IR	2.05 ± 1.39	2.73 ± 1.72	2.83 ± 1.91	3.92 ± 3.66	0.006
AST (U/L)	21.56 ± 7..75	23.63 ± 7.90	23.17 ± 9.69	27.96 ± 26.08	0.089
ALT (U/L)	20.66 ± 12.10	26.28 ± 16.72	30.27 ± 18.90	39.91 ± 30.06	0.001

OSA, obstructive sleep apnea; BMI, body mass index; AHI, apnea-hypopnea index; ODI, oxygen desaturation index; T90%, the percentage of total sleep time spent with SpO_2_ < 90%, LaSO_2_, lowest O_2_ saturation; ESS score, Epworth Sleepiness Scale score; TC, total cholesterol; TG, triglycerides; HDL-C, high-density lipoprotein-cholesterol; LDL-C, low-density lipoprotein-cholesterol; ALT, alanine aminotransferase; AST, aspartate aminotransferase

*p < 0.05

### Biochemical measurements

Blood samples were taken in the morning to measure the fasting serum concentrations of glucose, total cholesterol (TC), triglycerides (TG), high-density lipoprotein cholesterol (HDL-c), and low-density lipoprotein cholesterol (LDL-c). All parameters were analyzed with the H-7600 autoanalyzer (Hitachi, Tokyo, Japan). The homeostatic model assessment of insulin resistance (HOMA-IR) was calculated as fasting insulin (μIU/mL) × fasting glucose (mmol/L)/22.5 [13].

### Definition of NAFLD

Liver ultrasonography was performed for all participants using a 3.5-MHz transducer (Technos DU 8, Genoa, Italy) operated by an experienced radiologist who was blinded to the patients’ clinical data. Hepatic steatosis was diagnosed on the basis of characteristic ultrasonographic characteristics, i.e., evidence of diffuse hyper-echogenicity of the liver relative to the kidneys, ultrasound beam attenuation, and poor visualization of the intra-hepatic vessel borders and the diaphragm [14]. For evaluating moderate and severe steatosis, ultrasonography has high sensitivity and specificity, however, when the hepatic fat infiltration identified by liver biopsy is less than 30%, its sensitivity is reduced [15]. Semi-quantitative sonographic assessing the stage of hepatic steatosis was not carried out in this study. NAFLD was defined as the presence of fatty liver disease in the absence of excessive alcohol consumption.

### Statistical analysis

Data were analyzed using SPSS 19.0 (IBM Corp., Armonk, NY, USA). Before statistical analysis was performed, all descriptive data were tested for a normal distribution. Normally distributed data are represented as mean ± standard, deviation data are expressed as skewed median (interquartile range). In addition, categorical data are expressed as proportion (percentage). For normally distributed continuous variables, one-way analysis of variance (ANOVA) followed by post hoc comparison was performed to compare the data among the three groups. For data that were not normally distributed, the Kruskal–Wallis H test (K) was performed for multiple-group comparison. Categorical data were analyzed by the chi-square test or Fisher’s exact test. Stepwise logistic regression models were performed to evaluate independent risk factors for NAFLD. All *p* values were two-sided, and all results were considered statistically significant at *p* < 0.05.

## Results

In total, 415 subjects (aged 48.75 ± 23.98 y) were enrolled in this study, with population was stratified by AHI thresholds (based on polysomnography) and NAFLD diagnosis (based on hepatic ultrasound).

As shown in Table 1, the prevalence of NAFLD in the non-OSA, mild OSA, moderate OSA, and severe OSA groups was 37.21%, 69.09%, 68.34%, and 78.08%, respectively, and the difference was significant (*p* = 0.000). Age, the use of antidiabetic agents, fasting glucose, TC, TG, LDL-c, and HDL-c did not significantly differ among the groups (*p* > 0.05), as determined by ANOVA. However, BMI, WC, NC, ESS, ODI, TS90, and ALT elevated apparently with increased AHI, whereas LaSO_2_ and mean SpO_2_ reduced significantly. In addition, insulin and HOMA-IR significantly increased as the severity of OSA increased.

Table 2 shows that, compared with patients with OSA who did not have NAFLD (*n* = 96), patients with OSA who did have NAFLD (*n* = 276) had significantly higher BMI, WC, AHI, ODI, and glycometabolism indicators, including HbA1c, fasting glucose, insulin, and HOMA-IR (*p* < 0.05). However, there was no significant difference in lipid index values, including TC, TG, LDL-c, and HDL-c, between patients who had OSA with or without NAFLD.

**Table 2:** Comparison of clinical characteristics, laboratory parameters, and polysomnographic parameters in OSA patients with NAFLD and without NAFLD in OSA

	With NAFLD	Without NAFLD	*p*
*N* (%)	276 (74.19%)	96 (25.81%)	
Age (years)	48.97 ± 11.89	48.94 ± 18.98	0.174
Male sex, number (%)	229 (82.97%)	74 (77.08%)	0.337
Antilipimic agents (%)	5.3%	4.4%	0.868
Antidiabetic agents (%)	8.6%	4.7%	0.002
Antihypertensive agents (%)	28.2%	9.3%	0.010
BMI (kg/m^2^)	27.85 ± 6.66	25.35 ± 3.53	0.000
Neck circumference (cm)	38.50 ± 9.06	36.66 ± 8.84	0.081
Waist circumference (cm)	94.46 ± 22.26	88.94 ± 21.00	0.033
AHI	41.48 ± 23.30	33.78 ± 21.34	0.004
ODI	34.59 ± 24.00	26.44 ± 21.77	0.004
TS90%	9.64 ± 15.51	8.49 ± 22.39	0.580
LaSO_2_ (%)	70.42 ± 14.26	76.07 ± 12.59	0.000
mean SpO_2_ (%)	91.45 ± 7.30	92.91 ± 4.30	0.068
ESS score	9.08 ± 5.96	8.91 ± 5.68	0.804
HbA1c (%)	3.03 ± 3.24	1.52 ± 2.64	0.000
Fasting glucose (mmol/L)	5.58 ± 1.87	4.79 ± 1.35	0.000
Insulin (mmol/L)	73.76 ± 85.18	47.16 ± 47.45	0.000
TC (mmol/L)	5.14 ± 1.87	4.48 ± 1.53	0.182
TG (mmol/L)	3.53 ± 1.96	1.53 ± 1.30	0.369
LDL-c (mmol/L)	3.12 ± 1.18	2.85 ± 1.15	0.055
HDL-c (mmol/L)	1.09 ± 0.64	1.12 ± 0.43	0.674
HOMA-IR	3.78 ± 3.35	2.42 ± 1.57	0.000
AST (U/L)	21.56 ± 7.75	26.05 ± 20.92	0.174
ALT (U/L)	20.66 ± 12.10	35.34 ± 30.15	0.000

OSA, obstructive sleep apnea; BMI, body mass index; AHI, apnea-hypopnea index; ODI, oxygen desaturation index; T90%, the percentage of total sleep time spent with SpO_2_ < 90%; LaSO_2_, lowest O_2_ saturation; ESS score, Epworth Sleepiness Scale score; TC, total cholesterol; TG, triglycerides; HDL-C, high-density lipoprotein-cholesterol; LDL-C low-density lipoprotein-cholesterol; ALT, alanine aminotransferase; AST, aspartate aminotransferase

*p < 0.05

Finally, stepwise logistic regression suggested that TS90, LaSO_2_, and HOMA-IR were independent risk factors for NAFLD in all subjects after adjusting for potential confounding factors (odds ratio [OR] = 1.037, p = 0.014; OR = 1.056, *p* = 0.004; OR = 0.732, *p* = 0.009; respectively) (Table 3, Model 1). Furthermore, we found that TS90, LaSO_2_, and HOMA-IR were also independent risk factors for NAFLD in mild and moderate OSA patients, whereas TS90, LaSO_2_, and ODI were independent risk factors for NAFLD in severe OSA patients.

**Table 3:** Stepwise logistic regression analysis of factors identified risk factors for NAFLD

Factors	Odds ratio	95% CI	*p* values
Model 1			
TS90	1.037	(1.008, 1.068)	0.014*
LaSO_2_	1.056	(1.018, 1.096)	0.004*
HOMA-IR	0.732	(0.580, 0.924)	0.009*
Model 2			
TS90	1.040	(1.009, 1.073)	0.012*
LaSO_2_	1.064	(1.021, 1.108)	0.003*
HOMA-IR	0.727	(0.568, 0.932)	0.012*
Model 3			
TS90	1.056	(1.015, 1.097)	0.006*
LaSO_2_	1.053	(1.004, 1.104)	0.032*

Model 1 showed that TS90, LaSO2, and HOMA-IR were independent predictors of NAFLD in all subjects after adjusting body mass index, AHI, TS90%, LaSO2, mean SpO2, ODI, HOMA-IR, TC, triglycerides, and HDL-C. Model 2 and Model 3 showed TS90, LaSO_2_, and HOMA-IR were also independent predictors for NAFLD in patients with mild and moderate OSA and TS90, LaSO_2_, and ODI were independent predictors for NAFLD in patients with severe OSA respectively

OSA, obstructive sleep apnea; T90%, the percentage of total sleep time spent with SpO_2_ < 90%; LaSO_2_, lowest O_2_ saturation

*p < 0.05

## Discussion

It is widely recognized that NAFLD in patients with OSA remains an important disease burden, but the mechanisms underlying the effects of OSA on NAFLD are still unclear. Our study found that disordered glycometabolism and intermittent hypoxia can promote the development of NAFLD in patients with OSA. Furthermore, after adjusting for multiple variables, we found that intermittent hypoxia alone was an independent predictor for NAFLD in patients with severe OSA. Meanwhile, we did not observe any effect of lipid metabolism on the risk of development and progression of NAFLD.

Chronic intermittent hypoxia and sleep fragmentation induced by OSA have been considered to be associated with the development and progression of NAFLD [16]. Emerging data have confirmed that OSA contributed to an increased prevalence and severity of NAFLD, and a few studies have shown that treating OSA has a positive effect on transaminase elevation and radiological steatosis [17, 18]. Two recent meta-analyses have suggested that there is a relationship between NAFLD and OSA. Jin and his colleagues [10] analyzed nine studies (2272 participants) and found that the presence of OSA was associated with the presence of steatosis, lobular inflammation, ballooning degeneration, and fibrosis. Musso et al. [19] performed a similar meta-analysis and found that there was an association between OSA and the presence and the stage of NAFLD. They found that patients with OSA had a greater than twofold increased risk of developing NAFLD, and that patients with NAFLD who also had OSA had an approximately twofold higher risk of developing progressive NASH and fibrosis compared with those without OSA, independent of potential confounders. Growing evidence also suggests that chronic intermittent hypoxia induced by more severe OSA may contribute to NASH pathogenesis and the associated liver fibrosis [16, 20, 21]. Our study shows that the prevalence of NAFLD increased with increasing severity of OSA, and that hypoxia was an independent predictor for NAFLD in patients with OSA, especially those with severe OSA.

OSA-mediated aggravation of oxidative stress and systemic inflammation have been involved in NAFLD development in humans and in animal models [22–24], and OSA has also been strongly linked to metabolic syndrome, including hypertension, dyslipidemia, and insulin resistance, which promote NAFLD progression [25, 26]. Rodent studies including epidemiologic data [27, 28], an animal study [29], and an interventional study [30] indicate that OSA is an independent factor in the development of insulin resistance, which may predispose patients to liver steatosis progression [31]. Our study provides further evidence that TS90, LaSO_2_, and HOMA-IR are independent predictors for NAFLD, suggesting that insulin resistance and intermittent hypoxia contribute to the development of NAFLD. Some studies have demonstrated pathophysiological links between OSA and disordered glycometabolism, including sleep fragmentation and intermittent hypoxia induced by activating higher sympathetic functions [32], reducing the expression of glucose transporter 4 [33], and aggravating the dysregulated expression of glucagon-like peptide 1 [34]. Therefore, we speculated that the disordered glycometabolism induced by OSA constitutes the “first hit” in the development of NAFLD. In addition, after adjusting for multiple variables, we found that TS90, LaSO_2_, and ODI were associated with NAFLD in patients with severe OSA, which supports the hypothesis that oxidative stress might act as the “second hit” leading to the progression of NAFLD. Overall, our study confirms that disordered glycometabolism and oxidative stress, which are both induced by chronic intermittent hypoxia, may be linked with the pathogenesis of NAFLD.

In addition, we did not observe any interaction between lipid metabolism and NAFLD, although the lipid profile (including TC, TG, and LDL-c values) was elevated in the NAFLD group, which is consistent with our previous study [35]. This could be explained by differences in study design, or the indirect pathway between chronic intermittent hypoxia and gene expression in lipogenesis.

The present study has several limitations. Firstly, this study was conducted using a cross-sectional design which cannot show a causative relationship between OSA and NAFLD. Secondly, we excluded other chronic liver disease such as autoimmune hepatitis and Wilson disease, and by medical history rather than blood test. In addition, we did not match some confounding factors such as BMI for each group; in order to overcome this problem, multivariate analysis was performed. Another potential limitation is that NAFLD was diagnosed by abdominal ultrasonography instead of by biopsy. However, ultrasonography has acceptable accuracy and correlates well with histological findings. Various other non-invasive methods to assess NAFLD including Steato Test and Fibro Test have been validated to evaluate steatosis and liver fibrosis, but we initially did not measure α2-macroglobulin and haptoglobin which were components of Steato Test and Fibro Test.

In conclusion, our study showed that there is a relationship between OSA and NAFLD and that the disordered combination of glycometabolism and intermittent hypoxia contributes to the progression of NAFLD in patients with OSA. Additional large-scale, prospective studies are warranted to explore the impact of OSA on liver injury in non-obese adults.
